# Non-Coding RNAs in Glioma Microenvironment and Angiogenesis

**DOI:** 10.3389/fnmol.2021.763610

**Published:** 2021-11-03

**Authors:** Dongxue Li, Zhe Zhang, Chengyu Xia, Chaoshi Niu, Wenchao Zhou

**Affiliations:** ^1^Intelligent Pathology Institute, the First Affiliated Hospital of USTC, Division of Life Sciences and Medicine, University of Science and Technology of China, Hefei, China; ^2^Department of Neurosurgery, the First Affiliated Hospital of USTC, Division of Life Sciences and Medicine, University of Science and Technology of China, Hefei, China; ^3^Basic Medical College, Qingdao University, Qingdao, China; ^4^Department of Pathology, the First Affiliated Hospital of USTC, Division of Life Sciences and Medicine, University of Science and Technology of China, Hefei, China

**Keywords:** non-coding RNAs, glioblastoma, tumor microenvironment, tumor vascularization, anti-angiogenic therapy

## Abstract

Glioma, especially glioblastoma, is the most common and lethal brain tumor. In line with the complicated vascularization processes and the strong intratumoral heterogeneity, tumor-associated blood vessels in glioma are regulated by multiple types of cells through a variety of molecular mechanisms. Components of the tumor microenvironment, including tumor cells and tumor-associated stromata, produce various types of molecular mediators to regulate glioma angiogenesis. As critical regulatory molecules, non-coding RNAs (ncRNAs) inside cells or secreted to the tumor microenvironment play essential roles in glioma angiogenesis. In this review, we briefly summarize recent studies about the production, delivery, and functions of ncRNAs in the tumor microenvironment, as well as the molecular mechanisms underlying the regulation of angiogenesis by ncRNAs. We also discuss the ncRNA-based therapeutic strategies in the anti-angiogenic therapy for glioma treatment.

## Introduction

As early as 1971, Folkman proposed that the growth of solid tumors would be inhibited in the absence of angiogenesis and that both tumor growth and metastasis depended on the formation of new blood vessels. He pointed out that inhibition of angiogenesis was of great significance in tumor therapy (Folkman, 1974). Hanahan and Weinberg (2011) reported tumor angiogenesis as one of the top 10 characteristics of tumors, affirming that angiogenesis played a crucial role in tumor proliferation, invasion, and metastasis (Plate et al., 2012). Generally, neovascularization is essential for the growth of tumors with a diameter of more than 1–2 mm by providing nutrition and oxygen and eliminating metabolic wastes (Cao, 2009). So far, six patterns of angiogenesis in solid tumors have been recognized: sprouting angiogenesis, vasculogenesis, vascular co-selection, intussusceptive vascular growth, vasculogenic mimicry, and tumor stem cell transdifferentiation (Jain and Carmeliet, 2012).

Glioma is the most common and aggressive intracranial central nervous system tumor. As a highly vascularized tumor, the growth, invasion, and recurrence of glioma are all dependent on angiogenesis, resulting in a correlation between vascular density and the degree of malignancy and prognosis (Hanahan and Weinberg, 2011). In particular, glioblastoma (GBM), the most malignant glioma, has a large number of dysplastic new vessels and is the most vascularized brain tumor in humans (Dubois et al., 2014; Giusti et al., 2016; Quezada et al., 2018). Glioma is also featured by the heterogeneity of the tumor tissue. Within the tumor microenvironment composed of tumor cells, stromal cells, and non-cellular components, glioma angiogenesis is a complex process with diverse patterns that are regulated by multiple factors. As a category of critical regulatory molecules in the tumor microenvironment, ncRNAs are indispensable for the communication between diverse cell components and the strict regulation of the expression and secretion of angiogenic factors and cytokines during tumor angiogenesis.

## Types of ncRNAs Regulating Angiogenesis in The Glioma Microenvironment

With the development of high through-put sequencing methodology, the majority of DNA sequence in the human genome has been elucidated. The ENCODE (Encyclopedia of DNA Elements) project revealed that less than 2% of the mammalian genome encode messenger RNA, but at least 70% of the genome are able to produce transcripts of different sizes, mostly ncRNAs (Feingold et al., 2004). ncRNAs play important roles in regulating life activities such as DNA replication, transcription, RNA processing, translation, and protein functions. Functionally, ncRNAs can be grossly divided into two categories: housekeeping and regulatory ncRNAs (Hirayama and Shinozaki, 2010). The housekeeping ncRNAs are parts of the critical molecular machinery required for basic life activities, including transport RNA carrying amino acids, small nucleolar RNA guiding RNA modification and processing, and ribosomal RNA involved in protein synthesis. The regulatory ncRNAs, such as microRNA, long noncoding RNA (lncRNA), and circular RNA (circRNA), participate in multiple processes including tumor angiogenesis (Morris and Mattick, 2014).

### microRNAs

miRNAs are important small ncRNAs with a length of 20–22 nucleotides. Classically, miRNAs bind to Ago2 protein to form an RNA-induced silencing complex, which then recognizes and binds to the target mRNA by complementary base pairing, leading to the degradation of the target mRNA (Yang F. et al., 2017). Numerous studies have indicated the critical roles of miRNAs in glioma angiogenesis. For example, in gliomas relative to normal brains, miR-296 is elevated in endothelial cells and directly targets hepatocyte growth factor-regulated tyrosine kinase substrate (HGS). Because HGS mediates the degradation of VEGFR2 and PDGFRβ, miR-296 upregulates VEGFR2 and PDGFRβ to promote angiogenesis (Wuerdinger et al., 2008). Aside from miR-296, other miRNAs including miR-93 and miR-675-5p have been reported to promote glioma angiogenesis (Fang et al., 2011; Tchaicha et al., 2011; Lo Dico et al., 2016). In contrast, some miRNAs such as miR-124-3p, miRNA-205, and miR-128 may play inhibitory roles in regulating vascularization (Shi et al., 2012; Yue et al., 2012; Adlakha and Saini, 2014; Zhang et al., 2018). The miRNAs that have been reported to regulate angiogenesis in glioma are summarized in Table 1.

**Table 1:** miRNAs involved in the regulation of glioma angiogenesis.

miRNA	Mechanism of action	Effect on angiogenesis	References
miR-296	Target the hepatocyte growth factor-regulated tyrosine kinase substrate (HGS) mRNA, leading to decreased levels of HGS and thereby reducing HGS-mediated degradation of the growth factor receptors VEGFR2 and PDGFR beta.	Promote	Wuerdinger et al. (2008)
miR-93	Promote angiogenesis by suppressing, at least in part, integrin-beta 8 expression.	Promote	Fang et al. (2011) and Tchaicha et al. (2011)
miR-675-5p	Interact with HIF-1 alpha mRNA and the RNA Binding Protein HuR in hypoxia-induced responses.	Promote	Lo Dico et al. (2016)
miR-124-3p	miR-124-3p/NRP-1/GIPC1 pathway.	Inhibit	Zhang et al. (2018)
miRNA-205	Suppress expression of VEGF-A by directly interacting with the putative miRNA-205 binding site at the 3′-UTR.	Inhibit	Yue et al. (2012)
miR-128	Suppress p70S6K1 and its downstream signaling molecules such as HIF-1 and VEGF expression.	Inhibit	Shi et al. (2012) and Adlakha and Saini (2014)

### lncRNAs

lncRNAs are transcripts longer than 200 nucleotides without open reading frames (Zhao W. et al., 2019). The abnormal expression of functional lncRNAs in gliomas suggests the involvement of lncRNAs in the occurrence, development, and other malignant phenotypes of gliomas (Peng et al., 2018). lncRNAs may function through the following four main mechanisms. First, lncRNAs bind to and regulate the modification, stability, localization, and interaction of target proteins. The NF-κB-interacting lncRNA NKILA is significantly upregulated in gliomas, and higher NKILA levels are correlated with poorer patient prognosis. NKILA functions by upregulating HIF-1α expression and the activity of the hypoxia pathway to enhance the Warburg effect and glioma angiogenesis (Chen et al., 2020). Second, lncRNAs bind to long-stranded RNA molecules, including mRNAs, lncRNAs, pre-mRNAs, and pre-miRNAs, to regulate the stability and translation of bound RNAs. For example, ANKHD1 and LINC00346 are elevated, whereas ZNF655 is reduced in glioma-associated endothelial cells. ANKHD1 binds to and enhances the stability of LINC00346, which in turn promotes the degradation of ZNF655 mRNA. ZNF655 functions to target the promoter of ANKHD1. Thus the ANKD1/LINC00346/ZNF655 feedback loop regulates glioma angiogenesis (Yang et al., 2020). Third, lncRNAs bind to miRNAs and relieve their inhibitory effects on downstream target genes. lncRNA H19 plays an important role in GBM by up-regulating the expression of the angiogenic factor VASH2 through inhibition of miR-29a (Jia et al., 2016; Jiang et al., 2016). In addition, H19 promotes angiogenesis through the miR-342/Wnt5a/β-catenin axis and the miR-138/HIF-1α axis (Liu Z. Z. et al., 2020; Zhou et al., 2020). Finally, lncRNAs bind to genomic DNAs to regulate gene transcription. For instance, SLC26A4-AS1 recruits NFKB1 to promote NPTX1 transcription, which exerts anti-angiogenic effects on glioma cells (Li et al., 2021). Whereas many studies have shown the promotion of angiogenesis by several lncRNAs (NEAT, HULC, SNHG16, linc00667, SNHG15, PVT1, etc.) through various downstream pathways (Jia et al., 2016; Zhu et al., 2016; Ma et al., 2017a,b; Zhang et al., 2018; Wang C. et al., 2019; Wang D. et al., 2019; Xu H. et al., 2019; Chen et al., 2020; Liu Z. Z. et al., 2020; Yang et al., 2020; Zhou et al., 2020), lncRNAs may negatively regulate angiogenesis. For example, lncRNA SLC26A4-AS1 suppresses angiogenesis by upregulating NPTX1 *via* NFKB1 transcriptional factor (Li et al., 2021), while LINC00320 inhibits angiogenesis by downregulating NFKB1-mediated AQP9. (Chang et al., 2020). Table 2 summarizes the lncRNAs participating in the regulation of glioma angiogenesis through different mechanisms.

**Table 2:** lncRNAs involved in the regulation of glioma angiogenesis.

lncRNA	Mechanism of action	Effect on angiogenesis	References
lncRNA NKILA	Increase the expression level of HIF-1 alpha and activate the hypoxia pathway.	Promote	Chen et al. (2020)
lncRNA HULC	Regulate ESM-1 *via* the PI3K/Akt/mTOR signaling pathway.	Promote	Zhu et al. (2016)
lncRNA PAXIP1-AS1	Recruit transcription factor ETS1 to upregulate KIF14 expression.	Promote	Zhang et al. (2018) and Xu H. et al. (2019)
LINC00346	ANKHD1/LINC00346/ZNF655 feedback loop	Promote	Yang et al. (2020)
lncRNA H19	Bind to miR-29a that targets the 3′-UTR region of vasohibin 2 (VASH2).	Promote	Jia et al. (2016)
	Regulate Wnt5a/beta-Catenin pathway *via* targeting miR-342.	Promote	Liu Z. Z. et al. (2020) and Zhou et al. (2020)
	Regulate the miR-138/HIF-1 alpha/VEGF axis.	Promote	Liu Z. Z. et al. (2020) and Zhou et al. (2020)
lncRNA NEAT1	Inhibit the angiogenic Akt- FGF-2/TGF-beta/VEGF signaling through ceRNA effect of miR-194–5p and lncRNA NEAT1.	Promote	Wang C. et al. (2019)
SNHG16 and linc00667	USF1/SNHG16/miR-212–3p/ALDH1A1 and USF1/linc00667/miR-429/ALDH1A1 axis regulates the VM of glioma cells.	Promote	Wang D. et al. (2019)
lncRNA SNHG15	Regulate VEGFA and Cdc42 expression *via* miR-153.	Promote	Ma et al. (2017b)
lncRNA PVT1	Regulate Atg7 and Beclin1 expression *via* miR-186.	Promote	Ma et al. (2017a)
lncRNA SLC26A4-AS1	Promote NPTX1 transcriptional activity by recruiting NFKB1.	Inhibit	Li et al. (2021)
LINC00320	Downregulate NFKB1-mediated AQP9.	Inhibit	Chang et al. (2020)

### circRNAs

CircRNAs are a new family of ncRNAs found in all eukaryotic cells. CircRNA covalently forms a closed loop structure with neither poly (A) tail nor polarity of 5′–3′. Because the structure of circRNA occludes endogenous biomolecules, circRNA is resistant against exonuclease Rnase R digestion and miRNA-mediated non-classical degradation. The biogenesis of circRNAs is regulated by specific cis-acting elements and trans-acting factors, resulting in a tissue-specific and cell-specific expression pattern (Kristensen et al., 2019). Classically, circRNAs specifically bind to and adsorb miRNAs, thereby relieving the inhibitory effects of miRNAs on downstream target genes. For example, down-regulation of circ_002136 expression significantly inhibits the survival, migration, and tube formation of glioma endothelial cells. Circ_002136 functionally targets miR-138-5p to upregulate SOX13, which as a target gene of miR-138-5p directly associates with and activates SPON2 to promote angiogenesis (He Z. et al., 2019). Table 3 summarizes the circRNAs (cZNF292, cir-DICER1, circ-SHKBP1, and circSCAF11) involved in the regulation of angiogenesis in the glioma microenvironment (Yang et al., 2016; He et al., 2018; He Q. et al., 2019; Meng et al., 2019). Of note, so far the studies about circRNAs in glioma angiogenesis mainly focus on the classical regulation of miRNAs. However, circRNAs have the capacity to directly bind to proteins and affect their functions. In addition, circRNAs may be associated with pre-mRNA cleavage and ribosomal RNA maturation. Future studies may reveal the involvement of circRNAs in angiogenesis through these non-classical mechanisms.

**Table 3:** circRNAs involved in the regulation of angiogenesis in glioma.

circRNA	Mechanism of action	Effect on angiogenesis	References
cZNF292	Regulate the Wnt/beta-catenin signaling pathway	Promote	Yang et al. (2016)
circ_002136	FUS/circ_002136/miR-138–5p/SOX13 feedback loop	Promote	He Z. et al. (2019)
cir-DICER1	MOV10/circ-DICER1 / miR-103a-3p (miR-382–5p) / ZIC4 pathway	Promote	He Q. et al. (2019)
circ-SHKBP1	Regulate miR-544a/FOXP1 and miR-379/FOXP2 pathways	Promote	He et al. (2018)
circSCAF11	Regulate the miR-421/SP1/VEGFA axis	Promote	Meng et al. (2019)

## ncRNA-Related Intercellular Communications That Regulate Glioma Angiogenesis

**Figure 1:**
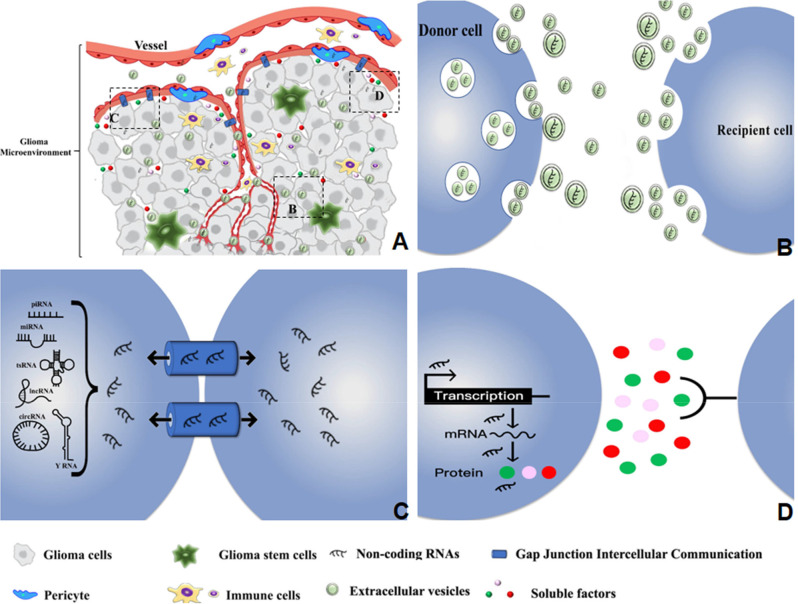
Diverse types of intercellular communications mediate the regulatory effects of ncRNAs on glioma angiogenesis in the tumor microenvironment. **(A)** The distribution and delivery of ncRNAs within the glioma microenvironment. **(B)** Delivery of ncRNAs from donor to recipient cells through the small EVs and medium/large EVs. **(C)** Transmission of ncRNAs between adjacent cells through GJIC based on gap junctions and channels. **(D)** ncRNA regulation of soluble factors that mediate the interaction between donor and recipient cells.

Cells and non-cellular components in the glioma microenvironment have complex communications that often favor tumor angiogenesis (Broekman et al., 2018). ncRNAs in the glioma microenvironment utilize multiple communication approaches to execute their functions as key regulators of angiogenesis (Figure 1).

### Soluble Factors

Numerous studies have emphasized the pivotal functions of soluble factors in angiogenesis, including the common proangiogenic factors VEGF, angiopoietin, and FGF, and the common angiogenic inhibitors arresten, endostatin, angiostatin, and MMP. Soluble factors are critical mediators of the functions of ncRNAs in regulating angiogenesis. For instance, the lncRNA PVT1 is able to bind and downregulate miR-26b and promote the expression of the soluble factors CTGF and ANGPT2 to boost vascularization (Zheng et al., 2018). CTGF is a member of the CCN family and other CCN proteins such as Cyr61 and NOV have similar functions in glioma (Le Mercier et al., 2008; Sin et al., 2008; Goodwin et al., 2010). Future studies may discover more ncRNAs for regulation of the CCN family. The most common proangiogenic factor VEGFA has been reported to be regulated by multiple ncRNAs including LINC01116, lncRNA CCAT2, and lncRNA TUG1 (Cai et al., 2017; Sun et al., 2020; Ye et al., 2020). It is reasonable that ncRNAs may regulate much more soluble factors to deliberately control angiogenesis (Yu et al., 2017; Yang et al., 2018). Table 4 summarizes the ncRNAs and their target soluble factors that regulate angiogenesis in the glioma microenvironment.

**Table 4:** ncRNAs and their target soluble factors involved in the regulation of angiogenesis in glioma.

ncRNA	Mechanism of action	Soluble factor	References
LINC01116	Compete with VEGFA for binding to miR-31–5p	VEGFA	Ye et al. (2020)
lncRNA CCAT2	Activate VEGFA signaling by sponging miR-424	VEGFA	Sun et al. (2020)
lncRNA TUG1	Directly bind to miR-299 to form an RNA-induced silencing complex	VEGFA	Cai et al. (2017)
lncRNA PVT1	Target miR-26b to activate CTGF/ANGPT2	CTGF/ANGPT2	Zheng et al. (2018)
lncRNA SBF2-AS1	Bind to miR-338-3p to regulate EGFL7 expression	EGFL7	Yu et al. (2017)
lncRNA MCM3AP-AS1	MCM3AP-AS1/miR211/KLF5/AGGF1 axis	AGGF1	Yang et al. (2018)

### The Gap Junction Intercellular Communication (GJIC)

During glioma invasion and progression, GJIC is an important intercellular communication process based on gap junctions and channels between two adjacent cells that connect the cytoplasm of cells (Katakowski et al., 2010; Hong et al., 2015). Briefly, six connexin (Cx) proteins assemble on one cell to form hemichannels (linkers) that pair with homotypic/heterotypic hemichannels of adjacent cells to mediate GJIC (Laird, 2006). miRNAs can cross gap junction channels to establish intercellular communication and directly affect gene expression in recipient cells (Lim et al., 2011). Cx43 is the most abundant connexin isoform in astrocytes, and down-regulation of Cx43 impairs tumor cell motility and invasiveness probably through disrupting the GJIC between astrocytes and tumor cells (Bates et al., 2007). GJIC also plays a role in functional miRNA transfer between GBM cells and human microvascular endothelial cells (HMEC; Thuringer et al., 2016). It has been demonstrated that miR-145-5p expressed in HMEC and miR-5096 derived from GBM cells can transfer between the two types of cells through GJIC. MiR-5096 may promote GBM invasiveness and angiogenesis after transferring into HMEC, while miR-145-5p acts as a tumor suppressor after moving into GBM cells (Thuringer et al., 2017). Direct cell-to-cell communication *via* GJIC is superior to indirect communication in terms of specificity and efficiency. in vivo delivery of therapeutic miRNAs through GJIC may be more effective but of a shorter duration than other delivery approaches (Lemcke et al., 2015).

### Extracellular Vesicles

Due to the lack of specific molecular markers, in 2018 the International Society for Extracellular Vesicles (ISEV) recommended stratifying extracellular vehicles (EV) into small vesicles <200 nm) and medium/large vesicles (>200 nm) according to their sizes (Thery et al., 2018). Several types of cells can secrete multiple types of EVs including exosomes (30–150 nm in diameter) derived from the inner vesicles, and microvesicles (100–1,000 nm in diameter) formed by outward budding of the cell membrane (Skog et al., 2008; Raposo and Stoorvogel, 2013). EVs play an important role in cell communication events such as direct cell-cell contact, plasma membrane fusion, and receptor-mediated endocytosis. Through transmitting the bioactive components of donor cells into recipient cells, EVs regulate the behaviors of recipient cells and affect the tumor microenvironment (Skog et al., 2008; Balaj et al., 2011; Montecalvo et al., 2012). The biomolecules as cargos of EVs include genomic DNA, cDNA, various RNAs, and proteins (cytoplasmic and membrane-bound), but ncRNAs are the most common and abundant contents of EVs that are transferred between cells with the strongest activity in regulating angiogenesis in the tumor microenvironment (Valadi et al., 2007; Van Der Vos et al., 2011; Rooj et al., 2016). A single GBM cell is able to secrete approximately 10,000 EVs in 48 h (Whitehead et al., 2020). Glioma cells express high levels of Lnc-HOTAIR that can be loaded into exosomes and transmitted to endothelial cells, which upregulates the pro-angiogenic factor VEGFA in vascular endothelial cells and ultimately enhances angiogenesis (Ma X. et al., 2017). Table 5 summarizes the ncRNAs enriched in EVs that regulate angiogenesis in the glioma microenvironment.

**Table 5:** ncRNAs in EVs from different cellular origins that regulate angiogenesis in glioma.

ncRNA	Cellular origins	Mechanism of action	References
miR-26	GSC	Activate the PI3K/Akt signaling pathway by targeting PTEN.	Wang Z.-F. et al. (2019)
miR-21	GSC	Stimulate miR-21/VEGF/VEGFR2 signal pathway in ECs	Sun et al. (2017) and Abels et al. (2019)
miR-9-5p	Glioma cell	Target RGS5, SOX7, and ABCB1	Lucero et al. (2020)
lnc-POU3F3	Glioma cell	Upregulate bFGF, bFGFR, VEGFA, and Angio in HBMECs	Lang et al. (2017)
miR-148a-3p	Glioma cell	Activate the EGFR/MAPK signaling pathway *via* inhibiting ERRFI1	Wang et al. (2020)
miR-1	Glioma cell	Inhibit JNK and Met	Boccaccio and Comoglio (2013))
	Glioma cell	Inhibit ANXA2	Gao et al. (2013)
lnc-HOTAIR	Glioma cell	Induction of VEGFA	Ma X. et al. (2017)
lncRNA-FTX	MSCs	Sponge miR-186 that binds to the 3′-UTR of c-Met	Liu L. et al. (2020)

## Cells in The Glioma Microenvironment Involved in ncRNA-Regulated Angiogenesis

### Glioma Cells and Glioma Stem Cells (GSCs)

ncRNAs may come from multiple cellular origins in the heterogeneous glioma microenvironment as summarized in Table 5. Among glioma cells, GSCs or glioma initiating cells are a small proportion of stem-like tumor cells with self-renewal and multi-lineage differentiation capacity. GSCs are enriched in perivascular niches and function as key regulatory cells to promote angiogenesis (Calabrese et al., 2007; Cheng et al., 2020). GSC-derived exosomes have a central role in angiogenesis and contain plenty ncRNAs in addition to pro-angiogenic factors (VEGF, TGF-β1, and CXCR4, etc.; Treps et al., 2017; Quezada et al., 2018). Skog et al. (2008) firstly reported that miRNAs loaded in EVs can be taken up by cultured endothelial cells to promote angiogenesis in GBMs. Recently, Lucero et al. (2020) performed RNA-seq on human brain endothelial cells exposed to GSC-derived EVs along with in silico analysis of epigenetic profiles of GBMs in the TCGA database, and they found that GSC-derived EVs activated transcription of multiple genes in endothelial cells that are highly correlated to angiogenesis. Of note, whereas some reports specifically studied the GSC-derived ncRNAs such as miR-26 and miR-21 in promoting angiogenesis (Sun et al., 2017; Abels et al., 2019; Wang Z.-F. et al., 2019), most studies did not emphasize the hierarchy of glioma cells when it comes to the cellular origin of ncRNAs (Boccaccio and Comoglio, 2013; Gao et al., 2013; Lang et al., 2017; Lucero et al., 2020; Wang et al., 2020). It remains an open question whether the expressions of proangiogenic ncRNAs are under the control, at least in part, of the signaling pathways specifically activated in GSCs.

### Mesenchymal Stem Cells

Glioma-associated mesenchymal stem cells (gbMSCs) are stromal cells associated with the malignant development of gliomas. gbMSCs consist of CD90^high^ and CD90^low^ subgroups with different roles in glioma progression. In particular, CD90^high^ gbMSCs function to increase proliferation and migration of glioma cells, while CD90^low^ gbMSCs have the capacity to transform into pericytes and alter miRNA expression profiles of vascular endothelial cells to promote angiogenesis (Yi et al., 2018). Intracranial xenografts derived from GSCs co-cultured with gbMSCs showed increased CD31 expression, suggesting that gbMSCs enhanced microvascular density (Kong et al., 2013). Because of the functional importance of MSCs in glioma angiogenesis, they may be both origins and targets of ncRNAs for the regulation of angiogenesis. So far there are very limited studies concerning ncRNAs in MSCs (Liu L. et al., 2020). Future studies about the ncRNAs in regulation of MSC phenotypes or the impact of MSC-derived ncRNAs on other components in tumor vasculature will certainly inspire the development of new anti-angiogenic strategies for glioma treatment.

### Immune Cells

The glioma tissues contain multiple types of immune cells, and tumor-associated macrophage (TAM) and microglia as the major glioma-infiltrating immune cells account for more than 30% of the GBM bulk cell population (Hambardzumyan et al., 2016). TAM is thought to be closer to macrophages of the M2 type and contributes to the resistance against anti-VEGF therapy through promoting angiogenesis and upregulating Treg cells (Zhu et al., 2017; Powell et al., 2018; Long et al., 2020). ncRNAs delivered by EVs and received by immune cells may be involved in angiogenesis. For example, miR-21 as a cargo of EV is delivered to and ingested by microglia, resulting in regulation of specific downstream mRNA targets and thus promoting angiogenesis (Abels et al., 2019). Future studies may discover more functions of glioma-infiltrating immune cells as the source cells and recipient cells of ncRNAs to regulate angiogenesis in the tumor microenvironment.

## Anti-Angiogenic Therapy by Targeting ncRNAs in The Glioma Microenvironment

Anti-angiogenic therapy has shown promising advances in treating many malignant tumors, but the current strategies are less optimistic in the treatment of gliomas (Carmeliet and Jain, 2011; Gilbert et al., 2014). Accumulating evidence has demonstrated that ncRNAs as key regulatory molecules are involved in the control of diverse target genes and multiple signaling pathways, thus participating in the dynamic and complex angiogenic process. Targeting ncRNAs in the glioma microenvironment will shed light on new strategies for the development of anti-angiogenic therapy.

### The Types of ncRNAs Utilized in RNA Therapy

RNA therapy refers to RNA-based targeted therapy either aiming at a specific RNA sequence or utilizing RNA-based molecules as drugs. RNA-based drugs can be grossly divided into siRNA/shRNA, miRNA, antisense oligonucleotide (ASO), and aptamer in terms of molecular structures. Relative to traditional small molecular chemical drugs and large molecular biopharmaceuticals, RNA therapy is more quantitative, dynamic, and flexible.

The majority of siRNA, miRNAs, and ASOs function by promoting the degradation or blocking the translation of target mRNAs. SiRNA, or small interfering RNA, is a class of double-stranded RNA with a length of 20–25 base pairs. By intraperitoneal injection of plasmids encoding siRNAs, Gondi et al. (2004, 2007) showed that siRNAs against uPA, the uPA receptor, and MMP-9 led to a significant reduction in glioma angiogenesis and tumor growth in preclinical mouse models. Likewise, Niola et al. (2006) showed that VEGF-targeted siRNA decreased GBM angiogenesis in a xenograft mouse model. Alternatively, Kargiotis et al. (2008) used adenovirus to deliver siRNA against MMP-2, resulting in decreased invasiveness and inhibition of angiogenesis. In addition to siRNAs, miRNAs and ASOs are being developed for cancer treatment. Both miRNA antagonists and miRNA mimics can work as miRNA-based drugs. As to glioma treatment, the miR-296 antagonist reduces angiogenesis in tumor xenografts *in vivo* (Wuerdinger et al., 2008). ASOs are small-sized single-stranded nucleic acids that form RNA-DNA hybrids with complementary RNA targets to promote RNase-H-mediated degradation of target genes. ASO had been used to target lncRNA TUG1 and inhibit the self-renewal of GSC (Katsushima et al., 2016).

Distinct from other types of RNA molecular drugs, aptamers are oligonucleotide or peptide molecules that bind to specific target molecules, also known as “chemical antibodies”. They are functionally comparable to traditional antibodies but have several advantages including small physical size, flexible structure, multifunctional chemical modification, high stability, and lack of immunogenicity. In addition, aptamers can be internalized after binding to certain receptors and self-cleaved in the presence of ribozyme and target molecules, making them useful as targeted delivery agents. Pegaptanib, approved by the FDA in 2004, is an RNA aptamer that selectively binds VEGF165 to prevent the interaction between VEGF165 and its receptors (VEGF-R1, VEGF-R2, Npn-1) and block VEGF165-mediated signaling, thereby inhibiting choroidal neovascularization and vascular leakage (Gragoudas et al., 2004; Stein and Castanotto, 2017).

Besides the aforementioned RNA-based drugs, targeting the physiological interaction between lncRNAs and miRNAs may provide new approaches for anti-angiogenic therapies (Zhao J. et al., 2019; Teppan et al., 2020). A large number of preclinical and clinical studies have demonstrated a bright future of RNA therapy with a broad spectrum of potential targets in tumor angiogenesis.

### EVs as Delivery Tools for RNA Therapy

EVs, especially exosomes, can work as privileged tools for the delivery of RNA cargos in the tumor microenvironment for angiogenic therapy of gliomas (Spinelli et al., 2019; Yekula et al., 2020). Compared with liposomal, metal, and polymeric nanomaterials, exosomes have better bioavailability but less cytotoxicity and immunogenicity. The membrane structure of EVs provides a stable and appropriate environment for the cargo molecules, and the signaling molecules on the surface of EV membranes may guide precise delivery to specific target cells. The ability of EVs to cross the blood-brain barrier is especially important for the treatment of brain cancers. Because of these advantages, EVs have strong potentials to be transformed into effective ncRNA delivery systems. A recent study showed that implantation of the miR-302–367-expressing donor cells to produce EVs containing miR-302–367 in the tumor microenvironment efficiently suppressed GBM development in mouse brain (Fareh et al., 2017; Nair et al., 2018). In addition, the EV donor cells may be genetically engineered to produce EVs with modified surfaces that could lead to more effective delivery to target cells. For example, the donor cells were engineered to express EGFR binding peptides on EV surface, and the modified EVs efficiently delivered let-7a specifically to EGFR-expressing tumor tissues (Ohno et al., 2013). Therefore, EVs may work as effective delivery tools for RNA therapy to express tumor-suppressive RNAs in the tumor microenvironment or specific tumor cells.

Despite the abovementioned advantages, the utilization of EVs in RNA therapy has been obstructed by several problems. There is no universally accepted standard for the isolation, purification, and preservation of exosomes. The mechanisms regulating the sorting and uptake of EVs in the tumor microenvironment remain unclear. Studies from different labs are somewhat incomparable because of the heterogeneity of EVs and the lack of a standardized quantification method. The application of EVs in RNA therapy requires a consensus on the methodology.

## The Emerging Future of ncRNAs in Angiogenesis

The rapidly developing RNA biology is unveiling the critical role of ncRNAs in angiogenesis from multiple unprecedented aspects. Not only new functions but also new categories of ncRNAs have been discovered in the past decade, which certainly will deepen our understanding of ncRNAs in the context of the glioma microenvironment.

### RNA Modification

More than one hundred RNA modifications have been reported so far. A well-known case is the N 6-methyladenosine (m6A) modification that refers to the reversible addition of a methyl group to the N element at position 6 of the A base in RNAs, which is the most abundant and well-characterized internal modification in mRNAs. Such RNA modifications occur also in regulatory ncRNAs, which may affect the protein-binding of lncRNAs, maturation of miRNAs, and translation of circRNAs to regulate their biogenesis and functions. The dynamic modifications may endow the ncRNAs with the flexibility to adapt to the constantly changing microenvironment and promote tumor progression.

Many research groups have observed the m6A modification in lncRNAs during the study of the polyA-enriched RNAs (Dominissini et al., 2012; Meyer et al., 2012). The m6A modification may affect the interaction between lncRNAs and their partner proteins. The lncRNA MALAT1 has m6A hypermethylation modification on multiple sites. Two of these m6A residues prevent the formation of the secondary structure of MALAT1 through the “m6A switch” mechanism and enhance the recognition and binding of hnRNPC to U5 channels in the MALAT1 hairpin (Dominissini et al., 2012; Liu et al., 2013, 2015). Interestingly, lncRNAs may also function as an m6A regulator. FOXM1-AS, a lncRNA antisense to FOXM1, promotes the interaction between the m6A demethylase ALKBH5 and the FOXM1 nascent transcripts, leading to the removal of m6A from FOXM1 transcripts to enhance FOXM1 expression in glioblastoma (Zhang et al., 2017).

The m6A modification participates in miRNA maturation. miRNA primary transcripts (pri-miRNA) transcribed from DNA undergo a series of cleavages to get into hairpin miRNA precursor (pre-miRNA) and finally mature miRNAs. However, the canonical m6A motif GGAC is abundant on pri-miRNAs but rarely seen on pre-miRNAs and mature miRNAs. The m6A writer METTL3 binds to pri-miRNAs to execute m6A modification. The m6A-bearing pri-RNAs are then recognized by hnRNPA2B1, which in turn interacts with the microRNA Microprocessor complex protein DGCR8 to promote pri-RNA processing. Therefore, alteration of METTL3 expression may impact the overall m6A levels and the expression of mature miRNAs (Alarcon et al., 2015a,b).

The m6A modification is also prevalent in circRNAs and its read-write mechanisms are similar to those in mRNAs. However, the functions of m6A modification in circRNAs may be different from those in mRNAs. The m6A reader YTHDF2 sequesters methylated circRNA to prevent the activation of the RNA pattern recognition receptor RIG-I, which is essential for the suppression of innate immunity (Zhou et al., 2017; Chen et al., 2019). Meanwhile, the m6A modifications in circRNAs seem to promote the translation initiation from circRNAs through YTHDF3 and the initiation factor eIF4G2 (Yang Y. et al., 2017).

Previous studies have shown that the m6A RNA methylase METTL3 promotes glioma progression by stabilizing the expression of SOX2, suggesting an important role of m6A modifications in glioma (Somasundaram, 2018; Visvanathan et al., 2018). To date, most studies have been focusing on the identification of m6A-modified mRNAs, while little is known about the m6A modification on ncRNAs. Future investigations about the impact of m6A modifications on the production, cellular location, target selection, and other features of ncRNAs will certainly inspire more explorations about the regulation of angiogenesis by ncRNAs in the glioma microenvironment.

### RNA-Binding Proteins

RNA-binding proteins (RBP) are a class of proteins that bind to RNAs, including mRNAs and ncRNAs, to regulate their biogenesis and expression levels (Xia et al., 2012; Xu Y. et al., 2019). The READDB^1^ database contains the information of 1,344 RBPs and the related diseases (Hashemikhabir et al., 2015). In addition, the catRAPID^2^ and the starBase v2.0^3^ databases provide useful tools to predict the interactions between RBPs and ncRNAs (Agostini et al., 2013; Li et al., 2014). The binding of RBP to ncRNAs participates in the regulation of target mRNAs from multiple aspects, including mRNA processing, maturation, transport, localization, and translation (Gerstberger et al., 2014; Janakiraman et al., 2018). In the meantime, the interaction between RBP and ncRNAs affects the expression levels of RBP and/or ncRNAs. Consequently, RBP-ncRNA interaction is involved in the development of neurodegenerative diseases, metabolic diseases, and various cancers (Kim et al., 2017).

RBP regulates not only the generation of miRNAs but also their functions. RBP may have antagonistic or facilitatory effects on miRNAs to synergistically regulate the translation of target genes (Van Kouwenhove et al., 2011; Ho and Marsden, 2014). RBP also affects the stability of lncRNAs. For example, the RBP IGF2BP1 binds to and destabilizes the lncRNA HULC in human hepatocellular carcinoma (Haemmerle et al., 2013). During the biosynthesis of circRNA, RBPs bind to introns near splicing sites and contribute to the production of circRNAs through the RBP-driven cyclization mechanism (Conn et al., 2015; Lyu and Huang, 2017). In addition, RBPs interact with circRNAs to regulate circRNA splicing, processing, folding, stabilization, and localization (Dudekulay et al., 2016). The roles of RBPs as upstream regulators of ncRNAs in glioma microenvironment and angiogenesis are largely unknown.

### The Diverse Types of ncRNAs

So far there is no available literature about ncRNAs other than miRNAs, lncRNAs, and circRNAs that have a role in glioma angiogenesis. However, accumulating reports emphasize the biological importance of the diverse types of ncRNAs, which may be star molecules for future studies on the glioma microenvironment.

#### piRNA

RNAs interacting with the PIWI protein, named as piRNAs (PIWI-interacting RNAs), are a class of 26–31 nt single-stranded small RNAs with a strong uridine monophosphate propensity at the 5’ end and methylation modification at the 3’ end, which are mainly present in mammalian germ cells and stem cells (Girard et al., 2006). piRNAs were first found in germ cells, functioning to repress transposons and maintain genome stability. Later studies about piRNAs in somatic and tumor cells highlight the diversity and importance of piRNAs. Human PIWI protein is abnormally expressed in breast, pancreatic, and liver cancers (Lee et al., 2006), and the Piwi-like family proteins are overexpressed in GBM (Huang et al., 2021). But little is known about the piRNAs in tumors. Based on the studies of piRNAs in the reproductive system, it is suggested that piRNAs may have similar functions as miRNAs and participate in tumor progression by regulating mRNA targets at the post-transcriptional level (Liu et al., 2019).

#### YRNA

YRNAs are a class of small RNAs with unique roles as signaling molecules in physiological and biochemical processes such as tumors and cardiovascular diseases, but the exact molecular functions of YRNAs remain elusive. YRNAs account for a relatively large proportion in tumor-derived exosomes, mainly distributed at 29–33 nt. There are four conserved types of YRNA transcripts: YRNA1 (hY1), YRNA3 (hY3), YRNA4 (hY4), and YRNA5 (hY5). In humans, the four YRNAs form a cluster at a single chromosomal locus on chromosome 7q36, which is transcribed by the RNA polymerase III. YRNAs consist of a stem-loop structure, an internal loop, and a polyuridine tail. The nucleotide sequences of the internal loop vary greatly, but the upper and lower domains are highly conserved among YRNAs. The loop domain is the least conserved element that regulates chromatin binding. Studies have shown that YRNAs function to initiate chromosomal DNA replication, which is related to cell proliferation (Gulia et al., 2020). Christov et al. (2008) found that the expression of all the four YRNAs was dramatically higher in solid tumors relative to corresponding normal tissues. Down-regulation of YRNA1 and YRNA3 resulted in significant inhibition of cell proliferation, suggesting that YRNA may be a new tumor biomarker and a therapeutic target. In gliomas, YRNA exists as a fragment of approximately 32 nt in length, and hY1, hY4, and hY5 are mainly found in exosomes or free RNP complexes (Wei et al., 2017).

#### tsRNA

The tRNA-derived small RNAs are collectively referred to as tsRNAs including tRNA-derived RNA fragment (tRF) and tRNA halves (tiRNA). TsRNAs have tissue- and cell-specific expression and are involved in a variety of biological functions, such as stress response, protein translation, ribosome biogenesis, intergenerational transmission of acquired epigenetic information, cell proliferation, apoptosis, and tumorigenesis (Tan et al., 2019). The levels of four tsRNAs (tRNA-ValTAC-3, tRNA-GlyTCC-5, tRNA-ValAAC-5, and tRNA-GluCTC-5) in plasma exosomes were significantly higher in HCC patients (Zhu et al., 2019). Serum tsRNA-ValTAC-41 and tsRNA-MetCAT-37 were used for the diagnosis of pancreatic ductal adenocarcinoma with high accuracy (Xue et al., 2021). The role of tsRNAs in glioma angiogenesis requires further exploration.

## Discussion

The ever-changing tumor microenvironment dominates angiogenesis from multiple aspects. ncRNAs are produced by a variety of donor cells to regulate multiple pathways in a series of different recipient cells in the tumor microenvironment to affect glioma angiogenesis. The complexity of the ncRNA regulatory network is both a chance and a challenge for the development of RNA therapy against angiogenesis. On one hand, ncRNA therapeutics can function on multiple target cells and multiple signaling pathways with high freedom to suppress angiogenesis. On the other hand, people should be super cautious to avoid unwanted off-target effects when utilizing ncRNAs. As for the drug delivery in ncRNA therapy, EVs may work as safe and effective tools to transport ncRNAs in the tumor microenvironment. However, the utilization of EVs requires a widely accepted standard system for the quality control of EVs. Of note, the naturally produced EVs as tumor supportive molecules may be important clinical targets for anti-angiogenic therapy. Taken together, ncRNAs as critical regulatory molecules in the tumor microenvironment are deeply involved in glioma angiogenesis. Future studies would certainly guarantee the anti-angiogenic therapy based on ncRNAs.

## Author Contributions

DL and WZ: conceptualization. WZ and CN: validation. DL and ZZ: writing—original draft preparation. WZ: writing—review and editing. CX and CN: supervision. DL, CN, and WZ: funding acquisition. All authors contributed to the article and approved the submitted version.

## Conflict of Interest

The authors declare that the research was conducted in the absence of any commercial or financial relationships that could be construed as a potential conflict of interest.

## Publisher’s Note

All claims expressed in this article are solely those of the authors and do not necessarily represent those of their affiliated organizations, or those of the publisher, the editors and the reviewers. Any product that may be evaluated in this article, or claim that may be made by its manufacturer, is not guaranteed or endorsed by the publisher.

## Footnotes

^1^ http://darwin.soic.iupui.edu/

^2^ http://s.tartaglialab.com/catrapid/omics

^3^ http://starbase.sysu.edu.cn/

## References

[B1] Abels E. R. Maas S. L. N. Nieland L. Wei Z. Cheah P. S. Tai E.. (2019). Glioblastoma-associated microglia reprogramming is mediated by functional transfer of extracellular miR-21. Cell Rep. 28, 3105–3119.e7. 10.1016/j.celrep.2019.08.036 31533034 PMC6817978

[B3] Adlakha Y. K. Saini N. (2014). Brain microRNAs and insights into biological functions and therapeutic potential of brain enriched miRNA-128. Mol. Cancer 13:33. 10.1186/1476-4598-13-33 24555688 PMC3936914

[B4] Agostini F. Zanzoni A. Klus P. Marchese D. Cirillo D. Gaetano Tartaglia G. (2013). catRAPID omics: a web server for large-scale prediction of protein-RNA interactions. Bioinformatics 29, 2928–2930. 10.1093/bioinformatics/btt495 23975767 PMC3810848

[B5] Alarcon C. R. Goodarzi H. Lee H. Liu X. Tavazoie S. Tavazoie S. F. (2015a). HNRNPA2B1 is a mediator of m(6)A-dependent nuclear RNA processing events. Cell 162, 1299–1308. 10.1016/j.cell.2015.08.011 26321680 PMC4673968

[B6] Alarcon C. R. Lee H. Goodarzi H. Halberg N. Tavazoie S. F. (2015b). N-6-methyladenosine marks primary microRNAs for processing. Nature 519, 482–485. 10.1038/nature14281 25799998 PMC4475635

[B7] Balaj L. Lessard R. Dai L. Cho Y. J. Pomeroy S. L. Breakefield X. O.. (2011). Tumour microvesicles contain retrotransposon elements and amplified oncogene sequences. Nat. Commun. 2:180. 10.1038/ncomms1180 21285958 PMC3040683

[B8] Bates D. C. Sin W. C. Aftab Q. Naus C. C. (2007). Connexin43 enhances glioma invasion by a mechanism involving the carboxy terminus. Glia 55, 1554–1564. 10.1002/glia.20569 17823969

[B9] Boccaccio C. Comoglio P. M. (2013). The MET oncogene in glioblastoma stem cells: implications as a diagnostic marker and a therapeutic target. Cancer Res. 73, 3193–3199. 10.1158/0008-5472.CAN-12-4039 23695554

[B10] Broekman M. L. Maas S. L. N. Abels E. R. Mempel T. R. Krichevsky A. M. Breakefield X. O. (2018). Multidimensional communication in the microenvirons of glioblastoma. Nat. Rev. Neurol. 14, 482–495. 10.1038/s41582-018-0025-8 29985475 PMC6425928

[B11] Cai H. Liu X. Zheng J. Xue Y. Ma J. Li Z.. (2017). Long non-coding RNA taurine upregulated 1 enhances tumor-induced angiogenesis through inhibiting microRNA-299 in human glioblastoma. Oncogene 36, 318–331. 10.1038/onc.2016.212 27345398

[B12] Calabrese C. Poppleton H. Kocak M. Hogg T. L. Fuller C. Hamner B.. (2007). A perivascular niche for brain tumor stem cells. Cancer Cell 11, 69–82. 10.1016/j.ccr.2006.11.020 17222791

[B13] Cao Y. H. (2009). Tumor angiogenesis and molecular targets for therapy. Front. Biosci. (Landmark Ed) 14, 3962–3973. 10.2741/3504 19273326

[B14] Carmeliet P. Jain R. K. (2011). Molecular mechanisms and clinical applications of angiogenesis. Nature 473, 298–307. 10.1038/nature10144 21593862 PMC4049445

[B15] Chang L. Bian Z. Xiong X. Liu J. Wang D. Zhou F.. (2020). Long non-coding RNA LINC00320 inhibits tumorigenicity of glioma cells and angiogenesis through downregulation of NFKB1-mediated AQP9. Front. Cell. Neurosci. 14:542552. 10.3389/fncel.2020.542552 33414706 PMC7782426

[B16] Chen Y. G. Chen R. Ahmad S. Verma R. Kasturi S. P. Amaya L.. (2019). N6-methyladenosine modification controls circular RNA immunity. Mol. Cell 76, 96–109.e9. 10.1016/j.molcel.2019.07.016 31474572 PMC6778039

[B17] Chen Z. Li S. Shen L. Wei X. Zhu H. Wang X.. (2020). NF-kappa B interacting long noncoding RNA enhances the Warburg effect and angiogenesis and is associated with decreased survival of patients with gliomas. Cell Death Dis. 11:323. 10.1038/s41419-020-2520-2 32382013 PMC7206073

[B18] Cheng J. Meng J. L. Zhu L. Peng Y. (2020). Exosomal noncoding RNAs in Glioma: biological functions and potential clinical applications. Mol. Cancer 19:66. 10.1186/s12943-020-01189-3 32213181 PMC7098115

[B20] Christov C. P. Trivier E. Krude T. (2008). Noncoding human Y RNAs are overexpressed in tumours and required for cell proliferation. Br. J. Cancer 98, 981–988. 10.1038/sj.bjc.6604254 18283318 PMC2266855

[B21] Conn S. J. Pillman K. A. Toubia J. Conn V. M. Salmanidis M. Phillips C. A.. (2015). The RNA binding protein quaking regulates formation of circRNAs. Cell 160, 1125–1134. 10.1016/j.cell.2015.02.014 25768908

[B22] Dominissini D. Moshitch-Moshkovitz S. Schwartz S. Salmon-Divon M. Ungar L. Osenberg S.. (2012). Topology of the human and mouse m(6)A RNA methylomes revealed by m(6)A-seq. Nature 485, 201–284. 10.1038/nature11112 22575960

[B23] Dubois L. G. Campanati L. Righy C. D’andrea-Meira I. De Sampaio E Spohr T. C. L. Porto-Carreiro I.. (2014). Gliomas and the vascular fragility of the blood brain barrier. Front. Cell. Neurosci. 8:418. 10.3389/fncel.2014.00418 25565956 PMC4264502

[B24] Dudekulay D. B. Panda A. C. Grammatikakis I. De S. Abdelmohsen K. Gorospe M. (2016). CircInteractome: a web tool for exploring circular RNAs and their interacting proteins and microRNAs. RNA Biol. 13, 34–42. 10.1080/15476286.2015.1128065 26669964 PMC4829301

[B25] Fang L. Deng Z. Shatseva T. Yang J. Peng C. Du W. W.. (2011). MicroRNA miR-93 promotes tumor growth and angiogenesis by targeting integrin-beta 8. Oncogene 30, 806–821. 10.1038/onc.2010.465 20956944

[B26] Fareh M. Almairac F. Turchi L. Burel-Vandenbos F. Paquis P. Fontaine D.. (2017). Cell-based therapy using miR-302–367 expressing cells represses glioblastoma growth. Cell Death Dis. 8:e2713. 10.1038/cddis.2017.117 28358371 PMC5386523

[B27] Feingold E. A. Good P. J. Guyer M. S. Kamholz S. Liefer L. Wetterstrand K.. (2004). The ENCODE (ENCyclopedia of DNA elements) project. Science 306, 636–640. 10.1126/science.1105136 15499007

[B28] Folkman J. (1974). Tumor angiogenesis factor. Cancer Res. 34, 2109–2113. 4842257

[B29] Gao H. Yu B. Yan Y. Shen J. Zhao S. Zhu J.. (2013). Correlation of expression levels of ANXA2, PGAM1 and CALR with glioma grade and prognosis. J. Neurosurg. 118, 846–853. 10.3171/2012.9.JNS112134 23082878

[B30] Gerstberger S. Hafner M. Tuschl T. (2014). A census of human RNA-binding proteins. Nat. Rev. Genet. 15, 829–845. 10.1038/nrg3813 25365966 PMC11148870

[B31] Gilbert M. R. Dignam J. J. Armstrong T. S. Wefel J. S. Blumenthal D. T. Vogelbaum M. A.. (2014). A randomized trial of bevacizumab for newly diagnosed glioblastoma. N. Engl. J. Med. 370, 699–708. 10.1056/NEJMoa1308573 24552317 PMC4201043

[B32] Girard A. Sachidanandam R. Hannon G. J. Carmell M. A. (2006). A germline-specific class of small RNAs binds mammalian piwi proteins. Nature 442, 199–202. 10.1038/nature04917 16751776

[B33] Giusti I. Delle Monache S. Di Francesco M. Sanita P. D’ascenzo S. Gravina G. L.. (2016). From glioblastoma to endothelial cells through extracellular vesicles: messages for angiogenesis. Tumour Biol. 37, 12743–12753. 10.1007/s13277-016-5165-0 27448307

[B34] Gondi C. S. Lakka S. S. Dinh D. H. Olivero W. C. Gujrati M. Rao J. S. (2004). Downregulation of uPA, uPAR and MMP-9 using small, interfering, hairpin RNA (siRNA) inhibits glioma cell invasion, angiogenesis and tumor growth. Neuron Glia Biol. 1, 165–176. 10.1017/s1740925x04000237 16804563 PMC1483066

[B35] Gondi C. S. Lakka S. S. Dinh D. H. Olivero W. C. Gujrati M. Rao J. S. (2007). Intraperitoneal injection of a hairpin RNA - Expressing plasmid targeting urokinase-type plasminogen activator (uPA) receptor and uPA retards angiogenesis and inhibits intracranial tumor growth in nude mice. Clin. Cancer Res. 13, 4051–4060. 10.1158/1078-0432.CCR-06-3032 17634529 PMC2139987

[B36] Goodwin C. R. Lal B. Zhou X. Ho S. Xia S. Taeger A.. (2010). Cyr61 mediates hepatocyte growth factor-dependent tumor cell growth, migration and Akt activation. Cancer Res. 70, 2932–2941. 10.1158/0008-5472.CAN-09-3570 20233866 PMC2848876

[B37] Gragoudas E. S. Adamis A. P. Cunningham E. T. Feinsod M. Guyer D. R. Neova V. I. S. O. (2004). Pegaptanib for neovascular age-related macular degeneration. N. Engl. J. Med. 351, 2805–2816. 10.1056/NEJMoa042760 15625332

[B38] Gulia C. Signore F. Gaffi M. Gigli S. Votino R. Nucciotti R.. (2020). Y RNA: an overview of their role as potential biomarkers and molecular targets in human cancers. Cancers (Basel) 12:1238. 10.3390/cancers12051238 32423154 PMC7281143

[B39] Haemmerle M. Gutschner T. Uckelmann H. Ozgur S. Fiskin E. Gross M.. (2013). Posttranscriptional destabilization of the liver-specific long noncoding RNA HULC by the IGF2 mRNA-binding protein 1 (IGF2BP1). Hepatology 58, 1703–1712. 23728852 10.1002/hep.26537

[B40] Hambardzumyan D. Gutmann D. H. Kettenmann H. (2016). The role of microglia and macrophages in glioma maintenance and progression. Nat. Neurosci. 19, 20–27. 10.1038/nn.4185 26713745 PMC4876023

[B41] Hanahan D. Weinberg R. A. (2011). Hallmarks of cancer: the next generation. Cell 144, 646–674. 10.1016/j.cell.2011.02.013 21376230

[B42] Hashemikhabir S. Neelamraju Y. Janga S. C. (2015). Database of RNA binding protein expression and disease dynamics (READ DB). Database (Oxford) 2015:bav072. 10.1093/database/bav072 26210853 PMC4515031

[B43] He Q. Zhao L. Liu X. Zheng J. Liu Y. Liu L.. (2019). MOV10 binding circ-DICER1 regulates the angiogenesis of glioma *via* miR-103a-3p/miR-382–5p mediated ZIC4 expression change. J. Exp. Clin. Cancer Res. 38:9. 10.1186/s13046-018-0990-1 30621721 PMC6323715

[B44] He Q. Zhao L. Liu Y. Liu X. Zheng J. Yu H.. (2018). circ-SHKBP1 regulates the angiogenesis of U87 glioma-exposed endothelial cells through miR-544a/FOXP1 and miR-379/FOXP2 pathways. Mol. Ther. Nucleic Acids 10, 331–348. 10.1016/j.omtn.2017.12.014 29499945 PMC5862134

[B45] He Z. Ruan X. Liu X. Zheng J. Liu Y. Liu L.. (2019). FUS/circ_002136/miR-138–5p/SOX13 feedback loop regulates angiogenesis in glioma. J. Exp. Clin. Cancer Res. 38:65. 10.1186/s13046-019-1065-7 30736838 PMC6368736

[B46] Hirayama T. Shinozaki K. (2010). Research on plant abiotic stress responses in the post-genome era: past, present and future. Plant J. 61, 1041–1052. 10.1111/j.1365-313X.2010.04124.x 20409277

[B47] Ho J. J. D. Marsden P. A. (2014). Competition and collaboration between RNA-binding proteins and microRNAs. Wiley Interdiscip. Rev. RNA 5, 69–86. 10.1002/wrna.1197 24124109

[B48] Hong X. Sin W. C. Harris A. L. Naus C. C. (2015). Gap junctions modulate glioma invasion by direct transfer of microRNA. Oncotarget 6, 15566–15577. 10.18632/oncotarget.3904 25978028 PMC4558171

[B49] Huang H. Yu X. Han X. Hao J. Zhao J. Bebek G.. (2021). Piwil1 regulates glioma stem cell maintenance and glioblastoma progression. Cell Rep. 34:108522. 10.1016/j.celrep.2020.108522 33406417 PMC7837390

[B50] Jain R. K. Carmeliet P. (2012). SnapShot: tumor angiogenesis. Cell 149, 1408–1408.e1. 10.1016/j.cell.2012.05.025 22682256

[B51] Janakiraman H. House R. P. Gangaraju V. K. Diehl J. A. Howe P. H. Palanisamy V. (2018). The long (lncRNA) and short (miRNA) of It: TGF beta-mediated control of RNA-binding proteins and noncoding RNAs. Mol. Cancer Res. 16, 567–579. 10.1158/1541-7786.MCR-17-0547 29555893 PMC6309266

[B52] Jia P. Cai H. Liu X. Chen J. Ma J. Wang P.. (2016). Long non-coding RNA H19 regulates glioma angiogenesis and the biological behavior of glioma-associated endothelial cells by inhibiting microRNA-29a. Cancer Lett. 381, 359–369. 10.1016/j.canlet.2016.08.009 27543358

[B53] Jiang X. C. Yan Y. K. Hu M. H. Chen X. D. Wang Y. X. Dai Y.. (2016). Increased level of H19 long noncoding RNA promotes invasion, angiogenesis and sternness of glioblastoma cells. J. Neurosurg. 124, 129–136. 10.3171/2014.12.JNS1426 26274999

[B54] Kargiotis O. Chetty C. Gondi C. S. Tsung A. J. Dinh D. H. Gujrati M.. (2008). Adenovirus-mediated transfer of siRNA against MMP-2 mRNA results in impaired invasion and tumor-induced angiogenesis, induces apoptosis *in vitro* and inhibits tumor growth *in vivo* in glioblastoma. Oncogene 27, 4830–4840. 10.1038/onc.2008.122 18438431 PMC2574662

[B55] Katakowski M. Buller B. Wang X. Rogiers T. Chopp M. (2010). Functional microRNA is transferred between glioma cells. Cancer Res. 70, 8259–8263. 10.1158/0008-5472.CAN-10-0604 20841486 PMC2970756

[B56] Katsushima K. Natsume A. Ohka F. Shinjo K. Hatanaka A. Ichimura N.. (2016). Targeting the notch-regulated non-coding RNA TUG1 for glioma treatment. Nat. Commun. 7:13616. 10.1038/ncomms13616 27922002 PMC5150648

[B57] Kim C. Kang D. Lee E. K. Lee J.-S. (2017). Long noncoding RNAs and RNA-binding proteins in oxidative stress, cellular senescence and age-related diseases. Oxid. Med. Cell Longev. 2017:2062384. 10.1155/2017/2062384 28811863 PMC5547732

[B58] Kong B. H. Shin H.-D. Kim S.-H. Mok H.-S. Shim J.-K. Lee J.-H.. (2013). Increased *in vivo* angiogenic effect of glioma stromal mesenchymal stem-like cells on glioma cancer stem cells from patients with glioblastoma. Int. J. Oncol. 42, 1754–1762. 10.3892/ijo.2013.1856 23483121

[B59] Kristensen L. S. Andersen M. S. Stagsted L. V. W. Ebbesen K. K. Hansen T. B. Kjems J. (2019). The biogenesis, biology and characterization of circular RNAs. Nat. Rev. Genet. 20, 675–691. 10.1038/s41576-019-0158-7 31395983

[B60] Laird D. W. (2006). Life cycle of connexins in health and disease. Biochem. J. 394, 527–543. 10.1042/BJ20051922 16492141 PMC1383703

[B61] Lang H. L. Hu G. W. Chen Y. Liu Y. Tu W. Lu Y. M.. (2017). Glioma cells promote angiogenesis through the release of exosomes containing long non-coding RNA POU3F3. Eur. Rev. Med. Pharmacol. Sci. 21, 959–972. 28338200

[B62] Le Mercier M. Mathieu V. Haibe-Kains B. Bontempi G. Mijatovic T. Decaestecker C.. (2008). Knocking down galectin 1 in human hs683 glioblastoma cells impairs both angiogenesis and endoplasmic reticulum stress responses. J. Neuropathol. Exp. Neurol. 67, 456–469. 10.1097/NEN.0b013e318170f892 18431251

[B63] Lee J. H. Schutte D. Wulf G. Fuzesi L. Radzun H. J. Schweyer S.. (2006). Stem-cell protein Piwil2 is widely expressed in tumors and inhibits apoptosis through activation of stat3/Bcl-X-L pathway. Hum. Mol. Genet. 15, 201–211. 10.1093/hmg/ddi430 16377660

[B64] Lemcke H. Steinhoff G. David R. (2015). Gap junctional shuttling of miRNA - a novel pathway of intercellular gene regulation and its prospects in clinical application. Cell Signal. 27, 2506–2514. 10.1016/j.cellsig.2015.09.012 26391653

[B65] Li H. Yan R. Chen W. Ding X. Liu J. Chen G.. (2021). Long non coding RNA SLC26A4-AS1 exerts antiangiogenic effects in human glioma by upregulating NPTX1 *via* NFKB1 transcriptional factor. FEBS J. 288, 212–228. 10.1111/febs.15325 32255252

[B66] Li J.-H. Liu S. Zhou H. Qu L.-H. Yang J.-H. (2014). starBase v2. 0: decoding miRNA-ceRNA, miRNA-ncRNA and protein-RNA interaction networks from large-scale CLIP-Seq data. Nucleic Acids Res. 42, D92–D97. 10.1093/nar/gkt1248 24297251 PMC3964941

[B67] Lim P. K. Bliss S. A. Patel S. A. Taborga M. Dave M. A. Gregory L. A.. (2011). Gap junction-mediated import of MicroRNA from bone marrow stromal cells can elicit cell cycle quiescence in breast cancer cells. Cancer Res. 71, 1550–1560. 10.1158/0008-5472.CAN-10-2372 21343399

[B68] Liu L. Li X. Shi Y. Chen H. (2020). The long noncoding RNA FTX promotes a malignant phenotype in bone marrow mesenchymal stem cells *via* the miR-186/c-Met axis. Biomed. Pharmacother. 131:110666. 10.1016/j.biopha.2020.110666 32853911

[B69] Liu N. Dai Q. Zheng G. He C. Parisien M. Pan T. (2015). N-6-methyladenosine-dependent RNA structural switches regulate RNA-protein interactions. Nature 518, 560–564. 10.1038/nature14234 25719671 PMC4355918

[B70] Liu N. Parisien M. Dai Q. Zheng G. He C. Pan T. (2013). Probing N-6-methyladenosine RNA modification status at single nucleotide resolution in mRNA and long noncoding RNA. RNA 19, 1848–1856. 10.1261/rna.041178.113 24141618 PMC3884656

[B71] Liu Y. Dou M. Song X. Dong Y. Liu S. Liu H.. (2019). The emerging role of the piRNA/piwi complex in cancer. Mol. Cancer 18:123. 10.1186/s12943-019-1052-9 31399034 PMC6688334

[B72] Liu Z. Z. Tian Y. F. Wu H. Ouyang S. Y. Kuang W. L. (2020). LncRNA H19 promotes glioma angiogenesis through miR-138/HIF-1 alpha/VEGF axis. Neoplasma 67, 111–118. 10.4149/neo_2019_190121N61 31777264

[B73] Lo Dico A. Costa V. Martelli C. Diceglie C. Rajata F. Rizzo A.. (2016). MiR675–5p Acts on HIF-1 alpha to sustain hypoxic responses: a new therapeutic strategy for glioma. Theranostics 6, 1105–1118. 10.7150/thno.14700 27279905 PMC4893639

[B74] Long Y. Tao H. Karachi A. Grippin A. J. Jin L. Chang Y.. (2020). Dysregulation of glutamate transport enhances treg function that promotes VEGF blockade resistance in glioblastoma. Cancer Res. 80, 499–509. 10.1158/0008-5472.CAN-19-1577 31723000

[B75] Lucero R. Zappulli V. Sammarco A. Murillo O. D. Cheah P. S. Srinivasan S.. (2020). Glioma-derived miRNA-containing extracellular vesicles induce angiogenesis by reprogramming brain endothelial cells. Cell Rep. 30, 2065–2074. 10.1016/j.celrep.2020.01.073 32075753 PMC7148092

[B76] Lyu D. Huang S. (2017). The emerging role and clinical implication of human exonic circular RNA. RNA Biol. 14, 1000–1006. 10.1080/15476286.2016.1227904 27588461 PMC5680672

[B77] Ma X. Li Z. H. Li T. Zhu L. W. S. Li Z. S. N. Tian N. (2017). Long non-coding RNA HOTAIR enhances angiogenesis by induction of VEGFA expression in glioma cells and transmission to endothelial cells *via* glioma cell derived-extracellular vesicles. Am. J. Transl. Res. 9, 5012–5021. 29218099 PMC5714785

[B78] Ma Y. Wang P. Xue Y. Qu C. Zheng J. Liu X.. (2017a). PVT1 affects growth of glioma microvascular endothelial cells by negatively regulating miR-186. Tumour. Biol. 39:1010428317694326. 10.1177/1010428317694326 28351322

[B79] Ma Y. Xue Y. Liu X. Qu C. Cai H. Wang P.. (2017b). SNHG15 affects the growth of glioma microvascular endothelial cells by negatively regulating miR-153. Oncol. Rep. 38, 3265–3277. 10.3892/or.2017.5985 29048682

[B80] Meng Q. Li S. Liu Y. Zhang S. Jin J. Zhang Y.. (2019). Circular RNA circSCAF11 accelerates the glioma tumorigenesis through the miR-421/SP1/VEGFA axis. Mol. Ther. Nucleic Acids 17, 669–677. 10.1016/j.omtn.2019.06.022 31400609 PMC6700438

[B81] Meyer K. D. Saletore Y. Zumbo P. Elemento O. Mason C. E. Jaffrey S. R. (2012). Comprehensive analysis of mRNA methylation reveals enrichment in 3 ’ UTRs and near stop codons. Cell 149, 1635–1646. 10.1016/j.cell.2012.05.003 22608085 PMC3383396

[B82] Montecalvo A. Larregina A. T. Shufesky W. J. Stolz D. B. Sullivan M. L. Karlsson J. M.. (2012). Mechanism of transfer of functional microRNAs between mouse dendritic cells *via* exosomes. Blood 119, 756–766. 10.1182/blood-2011-02-338004 22031862 PMC3265200

[B83] Morris K. V. Mattick J. S. (2014). The rise of regulatory RNA. Nat. Rev. Genet. 15, 423–437. 10.1038/nrg3722 24776770 PMC4314111

[B84] Nair K. G. S. Ramaiyan V. Sukumaran S. K. (2018). Enhancement of drug permeability across blood brain barrier using nanoparticles in meningitis. Inflammopharmacology 26, 675–684. 10.1007/s10787-018-0468-y 29582240

[B85] Niola F. Evangelisti C. Campagnolo L. Massalini S. Bue M. C. Mangiola A.. (2006). A plasmid-encoded VEGF siRNA reduces glioblastoma angiogenesis and its combination with interleukin-4 blocks tumor growth in a xenograft mouse model. Cancer Biol. Ther. 5, 174–179. 10.4161/cbt.5.2.2317 16340308

[B86] Ohno S. Takanashi M. Sudo K. Ueda S. Ishikawa A. Matsuyama N.. (2013). Systemically injected exosomes targeted to EGFR deliver antitumor microRNA to breast cancer cells. Mol. Ther. 21, 185–191. 10.1038/mt.2012.180 23032975 PMC3538304

[B87] Peng Z. Liu C. Wu M. (2018). New insights into long noncoding RNAs and their roles in glioma. Mol. Cancer 17:61. 10.1186/s12943-018-0812-2 29458374 PMC5817731

[B88] Plate K. H. Scholz A. Dumont D. J. (2012). Tumor angiogenesis and anti-angiogenic therapy in malignant gliomas revisited. Acta Neuropathologica 124, 763–775. 10.1007/s00401-012-1066-5 23143192 PMC3508273

[B89] Powell J. Mota F. Steadman D. Soudy C. Miyauchi J. T. Crosby S.. (2018). Small Molecule Neuropilin-1 Antagonists Combine Antiangiogenic and Antitumor Activity with Immune Modulation through Reduction of Transforming Growth Factor Beta (TGF beta) Production in Regulatory T-Cells. J. Med. Chem. 61, 4135–4154. 10.1021/acs.jmedchem.8b00210 29648813 PMC5957473

[B500] Quezada C. Torres A. Niechi I. Uribe D. Contreras-Duarte S. Toledo F.. (2018). extracellular vesicles in glioma progression. Mol. Aspects Med. 60, 38–51. 10.1016/j.mam.2017.12.003 29222067

[B90] Raposo G. Stoorvogel W. (2013). Extracellular vesicles: exosomes, microvesicles and friends. J. Cell Biol. 200, 373–383. 10.1083/jcb.201211138 23420871 PMC3575529

[B91] Rooj A. K. Mineo M. Godlewski J. (2016). MicroRNA and extracellular vesicles in glioblastoma: small but powerful. Brain Tumor Pathol. 33, 77–88. 10.1007/s10014-016-0259-3 26968172 PMC4853899

[B92] Shi Z.-M. Wang J. Yan Z. You Y.-P. Li C.-Y. Qian X.. (2012). MiR-128 inhibits tumor growth and angiogenesis by targeting p70S6K1. PLoS One 7:e32709. 10.1371/journal.pone.0032709 22442669 PMC3307714

[B93] Sin W. C. Bechberger J. F. Rushlow W. J. Naus C. C. (2008). Dose-dependent differential upregulation of CCN1/Cyr61 and CCN3/NOV by the gap junction protein connexin43 in glioma cells. J. Cell Biochem. 103, 1772–1782. 10.1002/jcb.21571 18004727

[B94] Skog J. Wurdinger T. Van Rijn S. Meijer D. H. Gainche L. Sena-Esteves M.. (2008). Glioblastoma microvesicles transport RNA and proteins that promote tumour growth and provide diagnostic biomarkers. Nat. Cell Biol. 10, 1470–1476. 10.1038/ncb1800 19011622 PMC3423894

[B95] Somasundaram K. (2018). N6-methyladenosine landscape of mRNAs in glioma: essential role of METTL3 and m(6)A modification in glioma stem cell growth. Cancer Med. 7, 17–17.

[B96] Spinelli C. Adnani L. Choi D. Rak J. (2019). Extracellular vesicles as conduits of non-coding RNA emission and intercellular transfer in brain tumors. Noncoding RNA 5:1. 10.3390/ncrna5010001 30585246 PMC6468529

[B97] Stein C. A. Castanotto D. (2017). FDA-approved oligonucleotide therapies in 2017. Mol. Ther. 25, 1069–1075. 10.1016/j.ymthe.2017.03.023 28366767 PMC5417833

[B98] Sun S.-L. Shu Y.-G. Tao M.-Y. (2020). LncRNA CCAT2 promotes angiogenesis in glioma through activation of VEGFA signalling by sponging miR-424. Mol. Cell Biochem. 468, 69–82. 10.1007/s11010-020-03712-y 32236863

[B99] Sun X. Ma X. Wang J. Zhao Y. Wang Y. Bihl J. C.. (2017). Glioma stem cells-derived exosomes promote the angiogenic ability of endothelial cells through miR-21/VEGF signal. Oncotarget 8, 36137–36148. 10.18632/oncotarget.16661 28410224 PMC5482644

[B100] Tan D.-M. Tan Y. Duan E.-K. (2019). Progress and prospect in tsRNA research. Prog. Biochem. Biophys. 46, 1063–1072. 10.16476/j.pibb.2019.0163

[B101] Tchaicha J. H. Reyes S. B. Shin J. Hossain M. G. Lang F. F. Mccarty J. H. (2011). Glioblastoma angiogenesis and tumor cell invasiveness are differentially regulated by beta 8 integrin. Cancer Res. 71, 6371–6381. 10.1158/0008-5472.CAN-11-0991 21859829 PMC3193578

[B102] Teppan J. Barth D. A. Prinz F. Jonas K. Pichler M. Klec C. (2020). Involvement of long non-coding RNAs (lncRNAs) in tumor angiogenesis. Noncoding RNA 6:42. 10.3390/ncrna6040042 32992718 PMC7711482

[B103] Thery C. Witwer K. W. Aikawa E. Alcaraz M. J. Anderson J. D. Andriantsitohaina R.. (2018). Minimal information for studies of extracellular vesicles 2018 (MISEV2018): a position statement of the international society for extracellular vesicles and update of the MISEV2014 guidelines. J. Extracell. Vesicles 7:1535750. 10.1080/20013078.2018.1535750 30637094 PMC6322352

[B104] Thuringer D. Boucher J. Jego G. Pernet N. Cronier L. Hammann A.. (2016). Transfer of functional microRNAs between glioblastoma and microvascular endothelial cells through gap junctions. Oncotarget 7, 73925–73934. 10.18632/oncotarget.12136 27661112 PMC5342024

[B105] Thuringer D. Solary E. Garrido C. (2017). The microvascular gap junction channel: a route to deliver microRNAs for neurological disease treatment. Front. Mol. Neurosci. 10:246. 10.3389/fnmol.2017.00246 28824376 PMC5543088

[B106] Treps L. Perret R. Edmond S. Ricard D. Gavard J. (2017). Glioblastoma stem-like cells secrete the pro-angiogenic VEGF-A factor in extracellular vesicles. J. Extracell. Vesicles 6:1359479. 10.1080/20013078.2017.1359479 28815003 PMC5549846

[B107] Valadi H. Ekstrom K. Bossios A. Sjostrand M. Lee J. J. Lotvall J. O. (2007). Exosome-mediated transfer of mRNAs and microRNAs is a novel mechanism of genetic exchange between cells. Nature Cell Biol. 9, 654–672. 10.1038/ncb1596 17486113

[B108] Van Der Vos K. E. Balaj L. Skog J. Breakefield X. O. (2011). Brain tumor microvesicles: insights into intercellular communication in the nervous system. Cell Mol. Neurobiol. 31, 949–959. 10.1007/s10571-011-9697-y 21553248 PMC3702172

[B109] Van Kouwenhove M. Kedde M. Agami R. (2011). MicroRNA regulation by RNA-binding proteins and its implications for cancer. Nat. Rev. Cancer 11, 644–656. 10.1038/nrc3107 21822212

[B110] Visvanathan A. Patil V. Arora A. Hegde A. S. Arivazhagan A. Santosh V.. (2018). Essential role of METTL3-mediated m(6)A modification in glioma stem-like cells maintenance and radioresistance. Oncogene 37, 522–533. 10.1038/onc.2017.351 28991227

[B111] Wang C. Chen Y. Wang Y. Liu X. Liu Y. Li Y.. (2019). Inhibition of COX-2, mPGES-1 and CYP4A by isoliquiritigenin blocks the angiogenic Akt signaling in glioma through ceRNA effect of miR-194–5p and lncRNA NEAT1. J. Exp. Clin. Cancer Res. 38:371. 10.1186/s13046-019-1361-2 31438982 PMC6704644

[B112] Wang D. Zheng J. Liu X. Xue Y. Liu L. Ma J.. (2019). Knockdown of USF1 inhibits the vasculogenic mimicry of glioma cells *via* stimulating SNHG16/miR-212–3p and linc00667/miR-429 Axis. Mol. Ther. Nucleic Acids 14, 465–482. 10.1016/j.omtn.2018.12.017 30743215 PMC6369224

[B113] Wang M. Zhao Y. Yu Z.-Y. Zhang R.-D. Li S.-A. Zhang P.. (2020). Glioma exosomal microRNA-148a-3p promotes tumor angiogenesis through activating the EGFR/MAPK signaling pathway *via* inhibiting ERRFI1. Cancer Cell Int. 20:518. 10.1186/s12935-020-01566-4 33117083 PMC7590612

[B114] Wang Z.-F. Liao F. Wu H. Dai J. (2019). Glioma stem cells-derived exosomal miR-26a promotes angiogenesis of microvessel endothelial cells in glioma. J. Exp. Clin. Cancer Res. 38:201. 10.1186/s13046-019-1181-4 31101062 PMC6525364

[B115] Wei Z. Batagov A. O. Schinelli S. Wang J. Wang Y. El Fatimy R.. (2017). Coding and noncoding landscape of extracellular RNA released by human glioma stem cells. Nat. Commun. 8:1145. 10.1038/s41467-017-01196-x 29074968 PMC5658400

[B116] Whitehead C. A. Kaye A. H. Drummond K. J. Widodo S. S. Mantamadiotis T. Vella L. J.. (2020). Extracellular vesicles and their role in glioblastoma. Crit. Rev. Clin. Lab. Sci. 57, 227–252. 10.1080/10408363.2019.1700208 31865806

[B117] Wuerdinger T. Tannous B. A. Saydam O. Skog J. Grau S. Soutschek J.. (2008). miR-296 regulates growth factor receptor overexpression in angiogenic endothelial cells. Cancer Cell 14, 382–393. 10.1016/j.ccr.2008.10.005 18977327 PMC2597164

[B118] Xia Z. Zheng X. Zheng H. Liu X. Yang Z. Wang X. (2012). Cold-inducible RNA-binding protein (CIRP) regulates target mRNA stabilization in the mouse testis. FEBS Lett. 586, 3299–3308. 10.1016/j.febslet.2012.07.004 22819822

[B119] Xu H. Zhao G. Zhang Y. Jiang H. Wang W. Zhao D.. (2019). Long non-coding RNA PAXIP1-AS1 facilitates cell invasion and angiogenesis of glioma by recruiting transcription factor ETS1 to upregulate KIF14 expression. J. Exp. Clin. Cancer Res. 38:486. 10.1186/s13046-019-1474-7 31823805 PMC6902534

[B120] Xu Y. Wu W. Han Q. Wang Y. Li C. Zhang P.. (2019). New insights into the interplay between non-coding RNAs and RNA-Binding protein HnRNPK in regulating cellular functions. Cells 8:62. 10.3390/cells8010062 30658384 PMC6357021

[B121] Xue M. Shi M. Xie J. Zhang J. Jiang L. Deng X.. (2021). Serum tRNA-derived small RNAs as potential novel diagnostic biomarkers for pancreatic ductal adenocarcinoma. Am. J. Cancer Res. 11, 837–848. 33791157 PMC7994152

[B122] Yang C. Zheng J. Liu X. Xue Y. He Q. Dong Y.. (2020). Role of ANKHD1/LINC00346/ZNF655 feedback loop in regulating the glioma angiogenesis *via* staufen1-mediated mRNA decay. Mol. Ther. Nucleic Acids 20, 866–878. 10.1016/j.omtn.2020.05.004 32464549 PMC7256448

[B123] Yang C. Zheng J. Xue Y. Yu H. Liu X. Ma J.. (2018). The effect of MCM3AP-AS1/miR-211/KLF5/AGGF1 axis regulating glioblastoma angiogenesis. Front. Mol. Neurosci. 10:437. 10.3389/fnmol.2017.00437 29375300 PMC5767169

[B124] Yang F. Ning Z. Ma L. Liu W. Shao C. Shu Y.. (2017). Exosomal miRNAs and miRNA dysregulation in cancer-associated fibroblasts. Mol. Cancer 16:148. 10.1186/s12943-017-0718-4 28851377 PMC5576273

[B125] Yang P. Qiu Z. Jiang Y. Dong L. Yang W. Gu C.. (2016). Silencing of cZNF292 circular RNA suppresses human glioma tube formation *via* the Wnt/beta-catenin signaling pathway. Oncotarget 7, 63449–63455. 10.18632/oncotarget.11523 27613831 PMC5325376

[B126] Yang Y. Fan X. Mao M. Song X. Wu P. Zhang Y.. (2017). Extensive translation of circular RNAs driven by N-6-methyladenosine. Cell Res. 27, 626–641. 10.1038/cr.2017.31 28281539 PMC5520850

[B127] Ye J. Zhu J. Chen H. Qian J. Zhang L. Wan Z.. (2020). A novel lncRNA-LINC01116 regulates tumorigenesis of glioma by targeting VEGFA. Int. J. Cancer 146, 248–261. 10.1002/ijc.32483 31144303

[B128] Yekula A. Yekula A. Muralidharan K. Kang K. Carter B. S. Balaj L. (2020). Extracellular vesicles in glioblastoma tumor microenvironment. Front. Immunol. 10:3137. 10.3389/fimmu.2019.03137 32038644 PMC6990128

[B129] Yi D. Xiang W. Zhang Q. Cen Y. Su Q. Zhang F.. (2018). Human glioblastoma-derived mesenchymal stem cell to pericytes transition and angiogenic capacity in glioblastoma microenvironment. Cell. Physiol. Biochem. 46, 279–290. 10.1159/000488429 29590646

[B130] Yu H. Zheng J. Liu X. Xue Y. Shen S. Zhao L.. (2017). Transcription factor NFAT5 promotes glioblastoma cell-driven angiogenesis *via* SBF2-AS1/miR-338–3p-Mediated EGFL7 expression change. Front. Mol. Neurosci. 10:301. 10.3389/fnmol.2017.00301 28983240 PMC5613209

[B131] Yue X. Wang P. Xu J. Zhu Y. Sun G. Pang Q.. (2012). MicroRNA-205 functions as a tumor suppressor in human glioblastoma cells by targeting VEGF-A. Oncol. Rep. 27, 1200–1206. 10.3892/or.2011.1588 22159356 PMC3583473

[B132] Zhang G. Chen L. Khan A. A. Li B. Gu B. Lin F.. (2018). miRNA-124–3p/neuropilin-1(NRP-1) axis plays an important role in mediating glioblastoma growth and angiogenesis. Int. J. Cancer 143, 635–644. 10.1002/ijc.31329 29457830

[B133] Zhang S. Zhao B. S. Zhou A. Lin K. Zheng S. Lu Z.. (2017). m(6)A demethylase ALKBH5 maintains tumorigenicity of glioblastoma stem-like cells by sustaining FOXM1 expression and cell proliferation program. Cancer Cell 31:591. 10.1016/j.ccell.2017.02.013 28344040 PMC5427719

[B135] Zhao J. Li L. Han Z.-Y. Wang Z.-X. Qin L.-X. (2019). Long noncoding RNAs, emerging and versatile regulators of tumor-induced angiogenesis. Am. J. Cancer Res. 9, 1367–1381. 31392075 PMC6682713

[B136] Zhao W. Shan B. He D. Cheng Y. Li B. Zhang C.. (2019). Recent progress in characterizing long noncoding RNAs in cancer drug resistance. J. Cancer 10, 6693–6702. 10.7150/jca.30877 31777598 PMC6856905

[B137] Zheng J. Hu L. Cheng J. Xu J. Zhong Z. Yang Y.. (2018). lncRNA PVT1 promotes the angiogenesis of vascular endothelial cell by targeting miR-26b to activate CTGF/ANGPT2. Int. J. Mol. Med. 42, 489–496. 10.3892/ijmm.2018.3595 29620147

[B138] Zhou C. Molinie B. Daneshvar K. Pondick J. V. Wang J. Van Wittenberghe N.. (2017). Genome-wide maps of m6A circRNAs identify widespread and cell-type-specific methylation patterns that are distinct from mRNAs. Cell Rep. 20, 2262–2276. 10.1016/j.celrep.2017.08.027 28854373 PMC5705222

[B139] Zhou Q. Liu Z.-Z. Wu H. Kuang W.-L. (2020). LncRNA H19 promotes cell proliferation, migration and angiogenesis of glioma by regulating Wnt5a/beta-catenin pathway *via* targeting miR-342. Cell. Mol. Neurobiol. [Online ahead of print]. 10.1007/s10571-020-00995-z 33161527 PMC11441209

[B140] Zhu C. Kros J. M. Cheng C. Mustafa D. (2017). The contribution of tumor-associated macrophages in glioma neo-angiogenesis and implications for anti-angiogenic strategies. Neuro Oncol. 19, 1435–1446. 10.1093/neuonc/nox081 28575312 PMC5737221

[B141] Zhu L. Li J. Gong Y. Wu Q. Tan S. Sun D.. (2019). Exosomal tRNA-derived small RNA as a promising biomarker for cancer diagnosis. Mol. Cancer 18:74. 10.1186/s12943-019-1000-8 30940133 PMC6444574

[B142] Zhu Y. Zhang X. Qi L. Cai Y. Yang P. Xuan G.. (2016). HULC long noncoding RNA silencing suppresses angiogenesis by regulating ESM-1 *via* the PI3K/Akt/mTOR signaling pathway in human gliomas. Oncotarget 7, 14429–14440. 10.18632/oncotarget.7418 26894862 PMC4924726

